# Scaling up integration: development and results of a participatory assessment of HIV/TB services, South Africa

**DOI:** 10.1186/1478-4505-8-23

**Published:** 2010-07-13

**Authors:** Vera Scott, Mickey Chopra, Virginia Azevedo, Judy Caldwell, Pren Naidoo, Brenda Smuts

**Affiliations:** 1 School of Public Health, University of the Western Cape, Modderdam Road, Bellville, Cape Town, 7535, South Africa; 2 Medical Research Council, Francie van Zijl Drive, Parowvallei, Cape Town, 7505, South Africa; 3 City Health, City of Cape Town, Cape Town, 7800, South Africa; 4 Department of Health, Provincial Government Western Cape, 44 Dorp Street, Cape Town, 7800, South Africa

## Abstract

**Background:**

In South Africa the need to integrate HIV, TB and STI programmes has been recognised at a policy and organisation level; the challenge is now one of translating policies into relevant actions and monitoring implementation to ensure that the anticipated benefits of integration are achieved. In this research, set in public primary care services in Cape Town, South Africa, we set out to determine how middle level managers could be empowered to monitor the implementation of an effective, integrated HIV/TB/STI service.

**Methods:**

A team of managers and researchers designed an evaluation tool to measure implementation of key components of an integrated HIV/TB/STI package with a focus on integration. They used a comprehensive health systems framework based on conditions for programme effectiveness and then identified and collected tracer indicators. The tool was extensively piloted in two rounds involving 49 clinics in 2003 and 2004 to identify data necessary for effective facility-level management. A subsequent evaluation of 16 clinics (2 per health sub district, 12% of all public primary care facilities) was done in February 2006.

**Results:**

16 clinics were reviewed and 635 records sampled. Client access to HIV/TB/STI programmes was limited in that 50% of facilities routinely deferred clients. Whilst the physical infrastructure and staff were available, there was problem with capacity in that there was insufficient staff training (for example, only 40% of clinical staff trained in HIV care). Weaknesses were identified in quality of care (for example, only 57% of HIV clients were staged in accordance with protocols) and continuity of care (for example, only 24% of VCT clients diagnosed with HIV were followed up for medical assessment). Facility and programme managers felt that the evaluation tool generated information that was useful to manage the programmes at facility and district level. On the basis of the results facility managers drew up action plans to address three areas of weakness within their own facility.

**Conclusions:**

This use of the tool which is designed to empower programme and facility managers demonstrates how engaging middle managers is crucial in translating policies into relevant actions.

## Background

With approximately 5 million people infected with HIV, South Africa faces a huge challenge in achieving improved health for all. The HIV epidemic is synergizing with a tuberculosis (TB) epidemic that was already well established. The estimated TB incidence (all forms) in South Africa has increased from 317 per 100 000 in 1995 to 948 per 100 000 in 2007 [1] with an estimated 73% of TB patients co-infected with HIV [2]. Cape Town is particularly affected with the double burden of TB and HIV. The antenatal prevalence of HIV has risen almost three-fold in recent years to reach more than 30% in some health districts [3]. The incidence of TB now exceeds 1 200 per 100 000 in some health sub districts [4].

The integration of the clinical and health systems management of HIV, TB and sexually transmitted infections (STIs) is attractive for clinicians and managers as it promises the possibility of increasing clinical and management efficiency [5]. WHO have identified key HIV/TB/STI interventions that should be offered depending upon the level of resources available [6]. This has been followed by policy guidelines [7] and the availability of increased resources. Greater partnership and collaboration between the different disease control programmes is seen as essential for successful integration. In particular the need to do joint planning, surveillance, monitoring and evaluation is emphasised [7]. In South Africa the feasibility and desirability of integrated HIV/TB service delivery have been tested locally with promising results [8,9]. South Africa has begun integration of key services with the clustering the HIV/AIDS and STI and TB Directorates in the Ministry of Health, the appointment of a national TB/HIV coordinating body, the recruitment of provincial TB/HIV coordinators and the development of integrated clinical guidelines. There is however some debate internationally as to whether integration does indeed deliver what it promises [10]. One systematic review of integration of vertical programmes concluded that there is no strong evidence of variation in the impact or outcome between vertically provided programmes and integrated ones [11]. This seems especially the case in resource-poor settings where there is a risk that resources will be spread so thinly across the different service-delivery activities and the support functions (such as supervision, logistics and training) that activities could fail to reach the minimum quantity and quality for any impact on health. Therefore there is a need for careful monitoring and evaluation to assess the implementation of integrated HIV/TB/STI service delivery. In particular the effect on programme performance at district and facility level is a sensitive indicator of whether the policy is achieving its goal of improving the quality and efficiency of services.

In Cape Town in 2002 a task team was established consisting of district programme managers and academics to address the research question: How can middle level health managers be empowered to monitor the implementation of an effective, integrated HIV/TB/STI service? In this paper we describe the development of a participatory monitoring and evaluation tool which, in the context of the prevailing fragmented HIV, TB and STI programmes, provided a uniform approach to quality assurance across the three programmes and introduced an integration lens within each programme to demonstrate missed opportunities in preventative, early case detection and care activities for the other programmes. We report on the 2006 results of a participatory evaluation using the tool and demonstrate how middle level managers were able to identify and address barriers to integrated HIV/TB/STI service delivery.

## Setting

Cape Town is a Metropolitan Municipality with 3.4 million inhabitants and is one of the 52 health districts in South Africa. For health administration, Cape Town is divided into 8 health sub districts, each with a population of around 420,000. There are 131 public primary care facilities - each facility has a facility manager who, together with the 8 health sub district managers they report to, represent the middle level of management in the health system. HIV, TB and STI services are offered at primary level through general rather than dedicated facilities. The HIV and TB programme managers at district level work closely with HIV/TB/STI (HAST) coordinators at sub district level who have a supportive supervisory role at facility level. At the time that this study was initiated, antiretroviral therapy was not part of the primary care package, although it has now been introduced as part of the general health services.

## Methods

**Figure 1:**
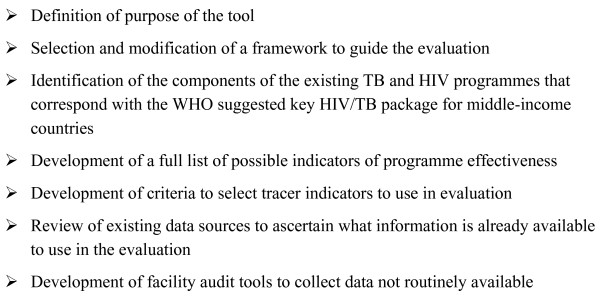
**Summary of key steps in the development of HIV/TB/STI evaluation tool**. ➢ Definition of purpose of the tool. ➢ Selection and modification of a framework to guide the evaluation. ➢ Identification of the components of the existing TB and HIV programmes that correspond with the WHO suggested key HIV/TB package for middle-income countries. ➢ Development of a full list of possible indicators of programme effectiveness. ➢ Development of criteria to select tracer indicators to use in evaluation. ➢ Review of existing data sources to ascertain what information is already available to use in the evaluation. ➢ Development of facility audit tools to collect data not routinely available

A task team consisting of three district programme managers who were responsible for HIV, TB and STIs, one sub district manager and two academics was formed to develop a process for integrating HIV and TB services, and comprised the core research team. Other district managers contributed on an ad hoc basis. This team prioritised the need to evaluate the existing programmes especially with a focus on the degree to which integration was occurring. They met on a monthly basis over 18 months to establish a monitoring and evaluation framework and develop appropriate indicators (Figure 1).

**Figure 2:**
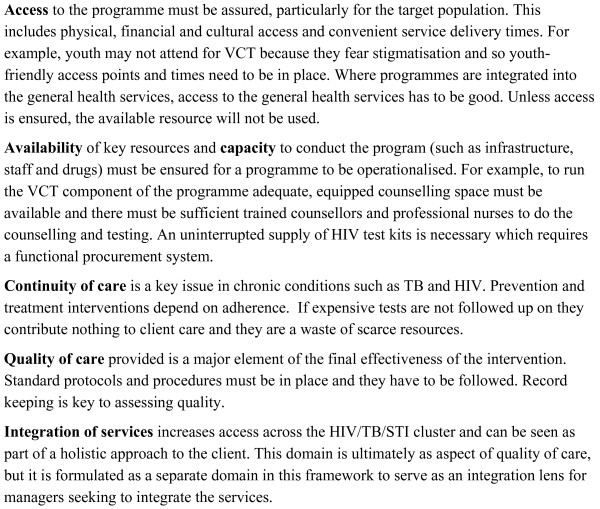
**Conditions of effectiveness**. **Access **to the programme must be assured, particularly for the target population. This includes physical, financial and cultural access and convenient service delivery times. For example, youth may not attend for VCT because they fear stigmatisation and so youth-friendly access points and times need to be in place. Where programmes are integrated into the general health services, access to the general health services has to be good. Unless access is ensured, the available resource will not be used. **Availability **of key resources and **capacity **to conduct the program (such as infrastructure, staff and drugs) must be ensured for a programme to be operationalised. For example, to run the VCT component of the programme adequate, equipped counselling space must be available and there must be sufficient trained counsellors and professional nurses to do the counselling and testing. An uninterrupted supply of HIV test kits is necessary which requires a functional procurement system. **Continuity of care **is a key issue in chronic conditions such as TB and HIV. Prevention and treatment interventions depend on adherence. If expensive tests are not followed up on they contribute nothing to client care and they are a waste of scarce resources. **Quality of care **provided is a major element of the final effectiveness of the intervention. Standard protocols and procedures must be in place and they have to be followed. Record keeping is key to assessing quality. **Integration of services **increases access across the HIV/TB/STI cluster and can be seen as part of a holistic approach to the client. This domain is ultimately as aspect of quality of care, but it is formulated as a separate domain in this framework to serve as an integration lens for managers seeking to integrate the services.

**Figure 3:**
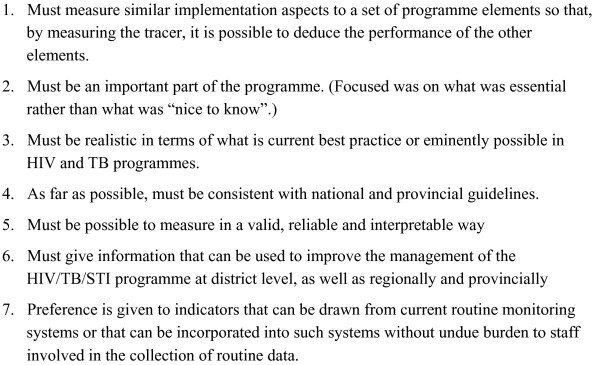
**Selection criteria for tracer indicators**. 1. Must measure similar implementation aspects to a set of programme elements so that, by measuring the tracer, it is possible to deduce the performance of the other elements. 2. Must be an important part of the programme. (Focused was on what was essential rather than what was "nice to know".) 3. Must be realistic in terms of what is current best practice or eminently possible in HIV and TB programmes. 4. As far as possible, must be consistent with national and provincial guidelines. 5. Must be possible to measure in a valid, reliable and interpretable way 6. Must give information that can be used to improve the management of the HIV/TB/STI programme at district level, as well as regionally and provincially 7. Preference is given to indicators that can be drawn from current routine monitoring systems or that can be incorporated into such systems without undue burden to staff involved in the collection of routine data.

The framework chosen for the tool was based on an expanded health systems approach which has been proposed by UNICEF/WHO for evaluating PMTCT programmes [12]. This framework is structured on the premise that for a programme to be effective a set of "critical conditions" [13] must be met. These critical conditions inform the domains that are then evaluated in the programmes performance: population targeting, access, availability of key resources, capacity, initial use of the service, quality of care and continuity of care. The team modified the UNICEF/WHO framework in three respects. Firstly, the domains population targeting and initial use where collapsed together under access. Secondly, availability of resources was merged with some measures of capacity. For example, the tool measures not just how many staff are available to do VCT (availability) but how many have been trained to offer VCT (capacity). Thirdly, a condition termed "integration" was added; in this domain managers specifically measured the extent to which the current HIV, TB and STI programmes were integrated. The five domains of the modified conditions of effectiveness framework are shown in Figure 2. Four key components of the integrated HIV/TB package recommended by WHO [6] were chosen for assessment: Voluntary Counselling and Testing (VCT), HIV medical care (excluding antiretrovirals), TB case detection and care and STI treatment. Aspects of the general service were also assessed, as this is the platform for programme delivery. Tracer indicators were identified and defined to assess the implementation and integration of the key programme components. We defined a tracer indicator as a focused measure of the performance of one aspect within a condition of effectiveness (or domain), that would allow managers to predict the likely programme performance across that domain. Tracer indicators were used because, in an evaluation of this magnitude and scope, it was not possible to measure all aspects of the programmes. Consensus on tracer indicators for each of the five conditions within each of the four key programme components was reached through the development of key selection criteria (Figure 3). Table 1 describes the final tracer indicators selected. A further exercise was conducted to identify a sub-set of indicators (termed "red flag indicators") which should be prioritised for management action if they were found to be areas of poor performance. This sub-set was limited to 3-5 indicators per programme; red flag indicators were selected because they measured requirements fundamental to the functioning of other aspects of the programme, or because they measured current district or programme priorities. Some of the tracer indicators could be calculated from existing routine data sources, such as the electronic TB register and the VCT registers. Other indicators required data elements that could only be collected through facility audits. Seven facility audit tools were developed. A facility manager interview schedule collected quantitative data regarding staffing levels and training, services delivered at the facility and facility systems. Two observation checklists for consulting and counselling rooms assessed whether these rooms were equipped to offer a quality consultation. A set of four folder reviews assessed the quality and continuity of care received by TB, STI, VCT and HIV positive clients. These tools are available from the authors on request.

**Table 1:** Tracer indicators of the Integrated HIV/TB/STI Evaluation Tool and 2006 audit results

Domain		Tracer indicator	Result (%)
Access	General	Facilities that routinely defer clients	50
		
		Facilities that routinely defer that have an appointment system	38
	
	HIV	Facilities HIV care offered daily	94
	
	TB	Facilities with triage to prioritise chronic cough	63
	
	STI	Facilities syndromic management of STIs offered daily	100
		
		Facilities with triage to prioritise STI's	44

Availability and Capacity	VCT	Lay counsellors trained in VCT counselling	98
		
		Clinical staff trained in VCT counselling	30
		
		Rooms equipped for quality counselling (private and stocked with dildos, condoms, IEC material)	45
	
	HIV	Clinical staff trained in HIV/AIDS	40
	
	STI	Clinical staff trained in Syndromic Mx	42
		
		Consulting rooms used to treat STI	42
		
		STI rooms fully equipped	12
	
	TB	Facilities with mechanism for recall of sputum positive clients	88
		
		Facilities with a dedicated TB nurse	100

Quality	VCT	Clients: Counselling forms used	90
		
		Clients: Consent for HIV test taken	91
		
		Clients: Safer sex was discussed	80
		
		Clients: Condoms were distributed	52
		
		Clients: Disclosure was discussed	70
	
	HIV	Clients: CD4 count done	81
		
		Clients: WHO staged	57
		
		Clients who are stage 4 OR CD4 < 200 who are referred for ARV treatment	68
	
	STI	Clients: Specific STI diagnosis made	81
		
		Clients: Correct drug regime used	81
		
		Clients: Clients offered condoms	71
		
		Clients: Clients given contact slips	71
		
		Clients: RPR done	84
	
	TB	Clients: Contact details complete	78
		
		Clients: Patient category is correct	96
		
		Clients: Sputum results adequately entered	81
		
		Clients: Patient is placed on the correct regimen	95
		
		Clients: Child contacts < 5yr assessed	35

Continuity	VCT	Positive clients attended for on-going counselling	24
		
		Positive clients attended for medical assessment	66
	
	HIV	Future management plan noted at last visit	61
	
	TB	New Smear Positive Interrupter rates	21
	
	STI	RPR results recorded in folder and acted on	84
	
Integration	VCT	Clients: Contraception discussed	34
		
		Clients: Screened for TB	70
		
		Clients: Screened for STI	68
	
	HIV	Clients: Contraception discussed	49
		
		Clients: Screened for TB at every visit	51
		
		Clients: Screened for STI every visit	79
		
		Female clients: PAP done	39
	
	STI	Clients: Contraception discussed	55

		Clients: offered VCT	71
	
	TB	Women: Contraception assessed	52
		
		Clients: VCT offered	94
		
		HIV + Clients prescribed bactrim	77
		
		HIV + Clients: CD4 count done	65

In February 2006 an assessment was conducted in 16 (12%) of the 131 public primary level facilities. Two facilities were selected from each of the 8 health sub districts. The task team wanted to give all sub district management teams exposure to the tools and process but limited the number of facilities so that individual facility-level support was feasible during the analysis process. Only facilities which indicated that they would be open to an evaluation process were considered. Four audit teams were formed which consisted of sub district and facility managers and HIV/TB/STI co-ordinators (three to four people per team). The teams received a full day of training on the rationale of the tool and the correct use for interviews, observations and folder reviews. Routine data were drawn from the routine data systems. The teams then visited the facilities on one day. In each facility the facility manager was interviewed, observations were done of the adequacy of equipment in counselling and consulting rooms and a random sample of VCT, HIV, TB and STI records was reviewed. Each facility visit took approximately 2 hours. Data were entered and analysed on a spreadsheet programme which calculated indicators and generated graphs of the results for each facility, as well as the aggregate results for the 16 facilities. On the second day all facility managers and district managers met in a workshop to review the results, to identify key constraints that needed to be addressed at facility level and to draw up action plans. Also in attendance were 2 to 3 staff members from each facility who were specifically identified by the facility manager as being able to support a follow-up quality improvement process in their facility.

Consent for the research was given by the two organisations delivering primary care services. Representatives from the primary care services in Cape Town were involved in their official capacity as programme managers and did so with a mandate from their organisation in accordance with their job descriptions which detail their role in monitoring and evaluating and quality improvement; they set the research agenda and agreed on the participatory methodology. The role of School of Public Health, University of the Western Cape, was to facilitate, advise on and document the process.

## Results

A total of 16 clinics were reviewed, 121 consulting rooms and 31 counselling rooms were inspected and 635 records were sampled. The full set of aggregated results for the 16 facilities is presented in Table 1. At the workshop on the second day managers and staff representatives validated the results, which increased their sense of ownership of the audit data. They then worked in staff teams led by each facility manager to identify the relative strengths and weaknesses of their individual facilities. Guided by the set of red flag indicators, three indicators of weakness were identified (across the 5 domains and the HIV/TB/STI programme components) for each facility and the possible reasons for these were discussed. Action plans were drawn up to implement strategies to improve the programme performance. Of particular interest was that many of the staff teams chose to promote integration of HIV, TB and STI services as a way of reducing missed opportunities for prevention and early case detection and improving performance.

Access in this setting was measured by whether facilities routinely deferred clients (i.e. turned clients away without assessment because the facility was deemed to be too busy to cope with the workload), whether they offered each programme component daily and whether assessment of clients presenting with symptoms suggestive of TB and STIs were prioritised. In this evaluation, although most facilities offered all the programme components, access was found to be limited in that 50% of facilities reported routinely deferring clients, only 44% prioritised the assessment of clients with STIs and 63% prioritised the assessment of clients presenting with a chronic cough. Further constraints to effective programme delivery were unpacked under the domain "Availability and Capacity" which measured availability of key resources, training of staff and whether process systems were in place, such as mechanisms to recall clients diagnosed with tuberculosis on the basis of positive sputa. The evaluation found that some key resources which in this setting were budgeted for by the district could be improved by strengthening facility level procurement and management skills. For example, of the consulting rooms used to treat STIs, only 12% were fully-equipped to enable staff to deliver a quality consultation: 48% had Syndromic Management Guidelines available, 26% had an adequate supply of speculae (as defined locally) and 59% had an adequate light source available. A further constraint which needed to be addressed at a higher organisational level was the inadequate level of staff training across the cluster of HIV/TB/STI: only 30% of clinical staff (professional nurses and doctors) were trained in VCT, 40% in general HIV care and 42% in syndromic management of STIs. All facilities had a dedicated, trained TB nurse.

**Figure 4:**
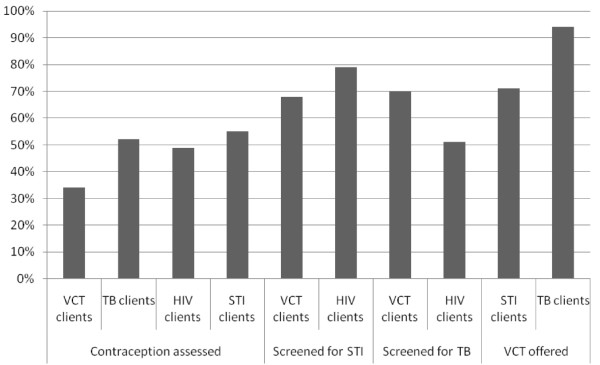
**Assessment of integration of HIV, TB and STI programmes**.

Quality of care was measured by assessing whether management guidelines were appropriately followed. The evaluation found that, once patients accessed the service (the first hurdle), quality of care was good in the TB programme but not in the HIV programme, possibly reflecting insufficient training coverage. Nearly all TB clients were correctly categorised (96%) and started on the correct drug regime (95%), but only 57% of HIV positive clients were staged according to WHO criteria (necessary for determining the correct management plan) and 68% were appropriately referred for anti-retroviral therapy in keeping with the then protocol. Continuity of care needed improvement across all programmes. Here measures looked at both clinical continuity in the care of clients and system processes to support continuity (such as functional record systems). There were many instances of folders which were requested for review not been found in the filing system. Only 24% of VCT clients who tested HIV positive received a follow up medical assessment. Ongoing management plans for clients receiving general HIV care were only recorded for 61% of clients. Register data showed that the smear positive TB interrupter rate (clients who interrupt treatment for 2 consecutive months divided by the total number of smear positive clients for the year) was 21% in 2005. The level of integration was variable but promising (generally over 65%) across the programmes. Staff were already implementing amended protocols which incorporated screening and early detection activities across the HIV/TB/STI cluster. Screening of VCT clients for TB and STIs was 70% and 68% respectively; screening of HIV clients for TB and STIs was 51% and 79% respectively; 71% of STI clients and 94% of TB clients were offered VCT (Figure 4). Assessment of clients for contraceptive needs was low across the programmes.

## Discussion

An earlier record-based assessment of TB and HIV services in one of the districts found little evidence of integration of services and concluded that important opportunities for increasing efficiency were being missed [8]. In contrast this study found that a significant proportion of clients were receiving integrated care. This was most evident in the TB programme where almost all patients were being offered VCT, whereas only half of the general HIV clients were being screened for TB. Overall, this study also found reasonable quality of care across all three programmes but there were important areas of concern. In particular the smear positive TB cure rate in Cape Town was less than 70%. From this participatory assessment with its additional integration lens middle level managers were able to engage in a data driven quality improvement process to build the individual programmes as well as to drive integration. Other HIV/TB programme evaluations [14,15] have tended to focus on the coverage of HIV/TB integration activities, rather than take a health systems approach to assessing the effectiveness of both programmes.

Programme managers are faced with a large amount of data that has to be collected and processed to satisfy national requirements and funder agreements. For example, Boerma and Stansfield [16] report that there are now more than 100 indicators that are expected from HIV/AIDS programmes alone. In this study the amount of data collected was rationalised by using a comprehensive health systems framework and key tracer indicators. We developed relatively simple data collection tools which enabled middle level managers to collect rapidly relevant data on the effectiveness of each individual programme component, as well as identify possible efficiency gains and missed opportunities where integration was not yet supported by policies and protocols. The drastically reduced amount of data allowed sub district, facility and programme managers to focus on the key aspects that influence effectiveness and to have more time for analysis and for planning appropriate interventions. The conceptual framework guided analysis and the red flags were useful in focusing attention on the most urgent problem areas. Overall, managers were able to collect, clean, analyse and prioritise actions for a sample of facilities within 2 days, enhancing the sustainability of the evaluation process. Subsequent to the audit process described here, the managers are now using the Integrated HIV/TB/STI evaluation tool to audit all 131 facilities twice a year as part of an internal quality improvement process, and it is now possible to track trends over time. The evaluation tool is also being used in the other 3 regions of the province and has been modified for use in rural districts in KwaZulu-Natal (Loveday, Scott, McCloughlin, Amien: Assessing and improving care for patients with TB/HIV/STIs in a rural district in KwaZulu-Natal South Africa, submitted) which has increased its generalisability: the tool is now widely applicable to both rural and urban South African public primary health settings.

The participatory process had certain limitations. Firstly, it was more time-consuming than an external evaluation process would have been and, secondly, it had to be driven by motivated and skilled supervisors and managers. A recent district-level participatory quality improvement intervention in South Africa to improve PMTCT coverage [17] identified poor supervision systems as a limitation to quality improvement and compensated by employing an external facilitator. In our setting this was not necessary as highly competent district programme managers drove the agenda. They recognised the key role of sub district and facility managers in programme implementation and quality improvement and so prioritised training and skill transfer throughout the evaluation process. A third limitation of the participatory process speaks to the same issue; the literature on quality improvement suggests that all evaluation tools and processes share a major limitation: they are only as good as the people who use them. This refers to people's skills and abilities, and their commitment to learning from the process. The quality of the relationship between levels of managers, supervisors and staff on the ground needs to be supportive and enabling for quality improvement [18].

The participatory process also had advantages. In this study it enabled facility and sub district managers to collect data from their own facilities which greatly increased their ownership of the data and their insight into the problems encountered at the facility and service provider level. The tool was a useful aid in orientating facility and sub district managers (whose main function is operational) to programmatic concerns. We found that facility managers at primary care level lacked problem-solving and action planning skills (even in their generic form). The participatory process allowed skill development in this area through the facilitated workshops. Individual mentoring was also required during the first few audit cycles. In subsequent audits, sub district HIV/TB/STI coordinators were able to take over much of the mentoring role, having developed their own skills during the earlier audit cycles. This skills transfer was necessary to allow for the evaluation process to be scaled up to include all 131 facilities. Supportive supervision is known to be able to improve service quality and provider performance [19,20]. Audit and feedback have been found to be effective as quality improvement processes (producing small to moderate improvements), but are not reliable as much depends on the context and manner in which they are undertaken [21].

Middle level managers face a complex task in running an effective HIV service, TB service and STI service. Integrating these services adds another order of complexity, requiring horizontal planning [22]. Experience of the shift in TB [23] and other programmes [24] from vertical to horizontal programming suggests that success is dependent upon maintaining the right mix between horizontal delivery at the health worker level (i.e. the health worker integrates HIV interventions with other interventions such as TB/STI) and the strengthening of management capacity at the district and provincial level to provide specific technical support. A global review of progress towards the millennium development goals has found that generic health-system interventions (e.g. management training, information system development etc.) have not impacted sufficiently on programme functioning and health outcomes [25]. In this participatory evaluation of an integrated HIV/TB/STI programme we have described a process located within the programme that supports the development of supervisory and management skills. There is evidence that the benefits of improved management within a programme can accrue to the health system as a whole, the so-called "diagonal approach" to health system strengthening [26].

## Conclusions

The participatory approach in developing and using the evaluation tools ensured that the information generated was relevant and useful to middle managers in managing the HIV, TB and STI programmes at district and facility level. It also enabled them to conceptualise how the integration of services could be achieved in their setting. The use of a health systems framework and tracer indicators rationalised the number of indicators collected. By using the data managers prioritised actions to improve service delivery by improving the quality of care and avoiding missed opportunities. Engaging middle managers is crucial in translating policies into relevant actions.

## Competing interests

The authors declare that they have no competing interests.

## Authors' contributions

VS and MC conceived of the overall evaluation approach and facilitated the development of the tool. VA, JC, PN, BS and VS designed the tools. VS and MC drafted the manuscript and VA, JC, PN, BS contributed through critical review. All the authors have read and approved the final manuscript.
